# Supplementary material for: Contrasting patterns of foraging behavior in neotropical stingless bees using pollen and honey metabarcoding
Source: Sci Rep. 2023 Sep 2;13:14474. doi: 10.1038/s41598-023-41304-0 (PMC10475120; doi:10.1038/s41598-023-41304-0)
Supplement: Supplementary file 1 — Supplementary Information 1. [file 41598_2023_41304_MOESM1_ESM.docx]

**Supplementary information**

**Figure S1.** Artificial colonies installed in the IBGE Reserve for the present study. A. Wooden box with a colony of *Scaptotrigona postica*. B. Inside the wood box, a colony of *Melipona rufiventris*.

**Figure S2.** Honey collection in the nests of (A) *Scaptotrigona postica* and (B) Melipona rufiventris

| **Table S1.** Pollen and honey sampling collected from either bees nests or bee body. Pollen and honey from nests were collected in several nests of three species: *Melipona rufiventris* (M), *Scaptotrigona postica* (S), *Tetragonisca angustula* (T). | | | | | |
| --- | --- | --- | --- | --- | --- |
| **Extraction number** | **Sample number** | **Bee species** | **Collecting date** | **Type** | **Date of DNA extraction** |
| 1 | 1 | *Tetragonisca angustula* | 21/9/2019 | pollen | 28/11/2019 |
| 2 | 2 | *Tetragonisca angustula* | 21/9/2019 | pollen | 28/11/2019 |
| 3 | 18 | *Scaptotrigona postica* | 11/10/2019 | pollen | 03/12/2019 |
| 3 | 19 | *Scaptotrigona postica* | 11/10/2019 | pollen | 03/12/2019 |
| 3 | 20 | *Scaptotrigona postica* | 11/10/2019 | pollen | 03/12/2019 |
| 4 | 27 | *Scaptotrigona postica* | 11/10/2019 | pollen | 03/12/2019 |
| 4 | 28 | *Scaptotrigona postica* | 11/10/2019 | pollen | 03/12/2019 |
| 4 | 29 | *Scaptotrigona postica* | 11/10/2019 | pollen | 03/12/2019 |
| 5 | 30 | *Scaptotrigona postica* | 11/10/2019 | pollen | 03/12/2019 |
| 5 | 31 | *Scaptotrigona postica* | 11/10/2019 | pollen | 03/12/2019 |
| 5 | 32 | *Scaptotrigona postica* | 11/10/2019 | pollen | 03/12/2019 |
| 6 | 36 | *Scaptotrigona postica* | 11/10/2019 | pollen | 03/12/2019 |
| 6 | 37 | *Scaptotrigona postica* | 11/10/2019 | pollen | 03/12/2019 |
| 6 | 38 | *Scaptotrigona postica* | 11/10/2019 | pollen | 03/12/2019 |
| 7 | 39 | *Tetragonisca angustula* | 11/10/2019 | pollen | 03/12/2019 |
| 7 | 40 | *Tetragonisca angustula* | 11/10/2019 | pollen | 03/12/2019 |
| 7 | 41 | *Tetragonisca angustula* | 11/10/2019 | pollen | 03/12/2019 |
| 8 | 42 | *Melipona rufiventris* | 11/10/2019 | pollen | 03/12/2019 |
| 8 | 44 | *Melipona rufiventris* | 11/10/2019 | pollen | 03/12/2019 |
| 8 | 45 | *Melipona rufiventris* | 11/10/2019 | pollen | 03/12/2019 |
| 9 | 52 | *Tetragonisca angustula* | 11/10/2019 | pollen | 03/12/2019 |
| 9 | 53 | *Tetragonisca angustula* | 11/10/2019 | pollen | 03/12/2019 |
| 9 | 54 | *Tetragonisca angustula* | 11/10/2019 | pollen | 03/12/2019 |
| 10 | 57 | *Tetragonisca angustula* | 11/10/2019 | pollen | 03/12/2019 |
| 10 | 58 | *Tetragonisca angustula* | 11/10/2019 | pollen | 03/12/2019 |
| 10 | 59 | *Tetragonisca angustula* | 11/10/2019 | pollen | 03/12/2019 |
| 11 | 64 | *Melipona rufiventris* | 11/10/2019 | pollen | 03/12/2019 |
| 12 | 67 | *Tetragonisca angustula* | 11/10/2019 | pollen | 03/12/2019 |
| 12 | 69 | *Tetragonisca angustula* | 11/10/2019 | pollen | 03/12/2019 |
| 13 | 70 | *Tetragonisca angustula* | 11/10/2019 | pollen | 03/12/2019 |
| 13 | 71 | *Tetragonisca angustula* | 11/10/2019 | pollen | 03/12/2019 |
| 13 | 72 | *Tetragonisca angustula* | 11/10/2019 | pollen | 03/12/2019 |
| 14 | 76 | *Scaptotrigona postica* | 11/10/2019 | pollen | 03/12/2019 |
| 14 | 77 | *Scaptotrigona postica* | 11/10/2019 | pollen | 03/12/2019 |
| 14 | 79 | *Scaptotrigona postica* | 11/10/2019 | pollen | 03/12/2019 |
| 15 | 84 | *Tetragonisca angustula* | 11/10/2019 | pollen | 04/12/2019 |
| 16 | 85 | *Scaptotrigona postica* | 11/10/2019 | pollen | 04/12/2019 |
| 17 | 87 | *Scaptotrigona postica* | 11/10/2019 | pollen | 04/12/2019 |
| 17 | 88 | *Scaptotrigona postica* | 11/10/2019 | pollen | 04/12/2019 |
| 17 | 89 | *Scaptotrigona postica* | 11/10/2019 | pollen | 04/12/2019 |
| 18 | 93 | *Scaptotrigona postica* | 29/10/2019 | pollen | 04/12/2019 |
| 18 | 94 | *Scaptotrigona postica* | 29/10/2019 | pollen | 04/12/2019 |
| 18 | 95 | *Scaptotrigona postica* | 29/10/2019 | pollen | 04/12/2019 |
| 19 | 99 | *Scaptotrigona postica* | 29/10/2019 | pollen | 04/12/2019 |
| 19 | 100 | *Scaptotrigona postica* | 29/10/2019 | pollen | 04/12/2019 |
| 19 | 101 | *Scaptotrigona postica* | 29/10/2019 | pollen | 04/12/2019 |
| 20 | 105 | *Tetragonisca angustula* | 29/10/2019 | pollen | 04/12/2019 |
| 20 | 106 | *Tetragonisca angustula* | 29/10/2019 | pollen | 04/12/2019 |
| 20 | 107 | *Tetragonisca angustula* | 29/10/2019 | pollen | 04/12/2019 |
| 21 | 111 | *Scaptotrigona postica* | 29/10/2019 | pollen | 04/12/2019 |
| 21 | 112 | *Scaptotrigona postica* | 29/10/2019 | pollen | 04/12/2019 |
| 22 | 120 | *Tetragonisca angustula* | 29/10/2019 | pollen | 04/12/2019 |
| 22 | 121 | *Tetragonisca angustula* | 29/10/2019 | pollen | 04/12/2019 |
| 22 | 122 | *Tetragonisca angustula* | 29/10/2019 | pollen | 04/12/2019 |
| 23 | 126 | *Melipona rufiventris* | 29/10/2019 | pollen | 04/12/2019 |
| 23 | 127 | *Melipona rufiventris* | 29/10/2019 | pollen | 04/12/2019 |
| 23 | 128 | *Melipona rufiventris* | 29/10/2019 | pollen | 04/12/2019 |
| 24 | 129 | *Melipona rufiventris* | 29/10/2019 | pollen | 04/12/2019 |
| 24 | 130 | *Melipona rufiventris* | 29/10/2019 | pollen | 04/12/2019 |
| 24 | 131 | *Melipona rufiventris* | 29/10/2019 | pollen | 04/12/2019 |
| 25 | 135 | *Tetragonisca angustula* | 29/10/2019 | pollen | 04/12/2019 |
| 25 | 136 | *Tetragonisca angustula* | 29/10/2019 | pollen | 04/12/2019 |
| 25 | 137 | *Tetragonisca angustula* | 29/10/2019 | pollen | 04/12/2019 |
| 26 | 144 | *Tetragonisca angustula* | 29/10/2019 | pollen | 04/12/2019 |
| 26 | 145 | *Tetragonisca angustula* | 29/10/2019 | pollen | 04/12/2019 |
| 26 | 146 | *Tetragonisca angustula* | 29/10/2019 | pollen | 04/12/2019 |
| 27 | 149 | *Tetragonisca angustula* | 29/10/2019 | pollen | 04/12/2019 |
| 27 | 151 | *Tetragonisca angustula* | 29/10/2019 | pollen | 04/12/2019 |
| 27 | 152 | *Tetragonisca angustula* | 29/10/2019 | pollen | 04/12/2019 |
| 28 | 156 | *Melipona rufiventris* | 29/10/2019 | pollen | 04/12/2019 |
| 28 | 157 | *Melipona rufiventris* | 29/10/2019 | pollen | 04/12/2019 |
| 28 | 158 | *Melipona rufiventris* | 29/10/2019 | pollen | 04/12/2019 |
| 29 | 159 | *Tetragonisca angustula* | 29/10/2019 | pollen | 04/12/2019 |
| 29 | 160 | *Tetragonisca angustula* | 29/10/2019 | pollen | 04/12/2019 |
| 29 | 161 | *Tetragonisca angustula* | 29/10/2019 | pollen | 04/12/2019 |
| 30 | 168 | *Tetragonisca angustula* | 29/10/2019 | pollen | 04/12/2019 |
| 30 | 169 | *Tetragonisca angustula* | 29/10/2019 | pollen | 04/12/2019 |
| 30 | 170 | *Tetragonisca angustula* | 29/10/2019 | pollen | 04/12/2019 |
| 31 | 174 | *Scaptotrigona postica* | 29/10/2019 | pollen | 04/12/2019 |
| 31 | 175 | *Scaptotrigona postica* | 29/10/2019 | pollen | 04/12/2019 |
| 31 | 176 | *Scaptotrigona postica* | 29/10/2019 | pollen | 04/12/2019 |
| 32 | 177 | *Scaptotrigona postica* | 29/10/2019 | pollen | 04/12/2019 |
| 32 | 178 | *Scaptotrigona postica* | 29/10/2019 | pollen | 04/12/2019 |
| 32 | 179 | *Scaptotrigona postica* | 29/10/2019 | pollen | 04/12/2019 |
| 33 | 186 | *Scaptotrigona postica* | 29/10/2019 | pollen | 04/12/2019 |
| 33 | 190 | *Scaptotrigona postica* | 29/10/2019 | pollen | 04/12/2019 |
| 33 | 191 | *Scaptotrigona postica* | 29/10/2019 | pollen | 04/12/2019 |
| 34 | 192 | *Tetragonisca angustula* | 12/11/2019 | pollen | 04/12/2019 |
| 34 | 193 | *Tetragonisca angustula* | 12/11/2019 | pollen | 04/12/2019 |
| 34 | 194 | *Tetragonisca angustula* | 12/11/2019 | pollen | 04/12/2019 |
| 35 | 198 | *Scaptotrigona postica* | 12/11/2019 | pollen | 04/12/2019 |
| 35 | 199 | *Scaptotrigona postica* | 12/11/2019 | pollen | 04/12/2019 |
| 35 | 200 | *Scaptotrigona postica* | 12/11/2019 | pollen | 04/12/2019 |
| 36 | 202 | *Scaptotrigona postica* | 12/11/2019 | pollen | 04/12/2019 |
| 36 | 203 | *Scaptotrigona postica* | 12/11/2019 | pollen | 04/12/2019 |
| 37 | 204 | *Scaptotrigona postica* | 12/11/2019 | pollen | 04/12/2019 |
| 37 | 205 | *Scaptotrigona postica* | 12/11/2019 | pollen | 04/12/2019 |
| 37 | 206 | *Scaptotrigona postica* | 12/11/2019 | pollen | 04/12/2019 |
| 38 | 212 | *Scaptotrigona postica* | 12/11/2019 | pollen | 04/12/2019 |
| 39 | 220 | *Tetragonisca angustula* | 12/11/2019 | pollen | 05/12/2019 |
| 39 | 221 | *Tetragonisca angustula* | 12/11/2019 | pollen | 05/12/2019 |
| 40 | 226 | *Melipona rufiventris* | 12/11/2019 | pollen | 05/12/2019 |
| 40 | 227 | *Melipona rufiventris* | 12/11/2019 | pollen | 05/12/2019 |
| 40 | 228 | *Melipona rufiventris* | 12/11/2019 | pollen | 05/12/2019 |
| 41 | 237 | *Tetragonisca angustula* | 12/11/2019 | pollen | 05/12/2019 |
| 41 | 238 | *Tetragonisca angustula* | 12/11/2019 | pollen | 05/12/2019 |
| 41 | 239 | *Tetragonisca angustula* | 12/11/2019 | pollen | 05/12/2019 |
| 42 | 240 | *Tetragonisca angustula* | 12/11/2019 | pollen | 05/12/2019 |
| 42 | 241 | *Tetragonisca angustula* | 12/11/2019 | pollen | 05/12/2019 |
| 42 | 250 | *Tetragonisca angustula* | 12/11/2019 | pollen | 05/12/2019 |
| 43 | 242 | *Tetragonisca angustula* | 12/11/2019 | pollen | 05/12/2019 |
| 43 | 243 | *Tetragonisca angustula* | 12/11/2019 | pollen | 05/12/2019 |
| 43 | 244 | *Tetragonisca angustula* | 12/11/2019 | pollen | 05/12/2019 |
| 44 | 254 | *Melipona rufiventris* | 12/11/2019 | pollen | 05/12/2019 |
| 44 | 255 | *Melipona rufiventris* | 12/11/2019 | pollen | 05/12/2019 |
| 44 | 256 | *Melipona rufiventris* | 12/11/2019 | pollen | 05/12/2019 |
| 45 | 260 | *Tetragonisca angustula* | 12/11/2019 | pollen | 05/12/2019 |
| 46 | 261 | *Scaptotrigona postica* | 12/11/2019 | pollen | 05/12/2019 |
| 46 | 262 | *Scaptotrigona postica* | 12/11/2019 | pollen | 05/12/2019 |
| 46 | 263 | *Scaptotrigona postica* | 12/11/2019 | pollen | 05/12/2019 |
| 47 | 267 | *Scaptotrigona postica* | 12/11/2019 | pollen | 05/12/2019 |
| 48 | 269 | *Tetragonisca angustula* | 12/11/2019 | pollen | 05/12/2019 |
| 48 | 270 | *Tetragonisca angustula* | 12/11/2019 | pollen | 05/12/2019 |
| 49 | 271 | *Scaptotrigona postica* | 12/11/2019 | pollen | 05/12/2019 |
| 49 | 272 | *Scaptotrigona postica* | 12/11/2019 | pollen | 05/12/2019 |
| 49 | 273 | *Scaptotrigona postica* | 12/11/2019 | pollen | 05/12/2019 |
| 50 | 279 | *Scaptotrigona postica* | 12/11/2019 | pollen | 05/12/2019 |
| 50 | 287 | *Scaptotrigona postica* | 12/11/2019 | pollen | 05/12/2019 |
| 50 | 288 | *Scaptotrigona postica* | 12/11/2019 | pollen | 05/12/2019 |
| 50 | 290 | *Scaptotrigona postica* | 12/11/2019 | pollen | 05/12/2019 |
| 51 | 295 | *Scaptotrigona postica* | 22/11/2019 | pollen | 06/12/2019 |
| 51 | 296 | *Scaptotrigona postica* | 22/11/2019 | pollen | 06/12/2019 |
| 51 | 297 | *Scaptotrigona postica* | 22/11/2019 | pollen | 06/12/2019 |
| 52 | 301 | *Scaptotrigona postica* | 22/11/2019 | pollen | 06/12/2019 |
| 52 | 310 | *Scaptotrigona postica* | 22/11/2019 | pollen | 06/12/2019 |
| 52 | 311 | *Scaptotrigona postica* | 22/11/2019 | pollen | 06/12/2019 |
| 53 | 304 | *Scaptotrigona postica* | 22/11/2019 | pollen | 06/12/2019 |
| 53 | 305 | *Scaptotrigona postica* | 22/11/2019 | pollen | 06/12/2019 |
| 53 | 306 | *Scaptotrigona postica* | 22/11/2019 | pollen | 06/12/2019 |
| 54 | 316 | *Tetragonisca angustula* | 22/11/2019 | pollen | 06/12/2019 |
| 54 | 317 | *Tetragonisca angustula* | 22/11/2019 | pollen | 06/12/2019 |
| 54 | 318 | *Tetragonisca angustula* | 22/11/2019 | pollen | 06/12/2019 |
| 54 | 369 | *Tetragonisca angustula* | 22/11/2019 | pollen | 06/12/2019 |
| 55 | 323 | *Tetragonisca angustula* | 22/11/2019 | pollen | 06/12/2019 |
| 55 | 324 | *Tetragonisca angustula* | 22/11/2019 | pollen | 06/12/2019 |
| 55 | 325 | *Tetragonisca angustula* | 22/11/2019 | pollen | 06/12/2019 |
| 56 | 329 | *Tetragonisca angustula* | 22/11/2019 | pollen | 06/12/2019 |
| 56 | 330 | *Tetragonisca angustula* | 22/11/2019 | pollen | 06/12/2019 |
| 56 | 331 | *Tetragonisca angustula* | 22/11/2019 | pollen | 06/12/2019 |
| 57 | 337 | *Melipona rufiventris* | 22/11/2019 | pollen | 06/12/2019 |
| 57 | 338 | *Melipona rufiventris* | 22/11/2019 | pollen | 06/12/2019 |
| 57 | 339 | *Melipona rufiventris* | 22/11/2019 | pollen | 06/12/2019 |
| 58 | 340 | *Tetragonisca angustula* | 22/11/2019 | pollen | 06/12/2019 |
| 58 | 354 | *Tetragonisca angustula* | 22/11/2019 | pollen | 06/12/2019 |
| 58 | 371 | *Tetragonisca angustula* | 22/11/2019 | pollen | 06/12/2019 |
| 59 | 341 | *Tetragonisca angustula* | 22/11/2019 | pollen | 06/12/2019 |
| 59 | 343 | *Tetragonisca angustula* | 22/11/2019 | pollen | 06/12/2019 |
| 59 | 352 | *Tetragonisca angustula* | 22/11/2019 | pollen | 06/12/2019 |
| 60 | 353 | *Tetragonisca angustula* | 22/11/2019 | pollen | 06/12/2019 |
| 61 | 355 | *Melipona rufiventris* | 22/11/2019 | pollen | 06/12/2019 |
| 61 | 357 | *Melipona rufiventris* | 22/11/2019 | pollen | 06/12/2019 |
| 61 | 358 | *Melipona rufiventris* | 22/11/2019 | pollen | 06/12/2019 |
| 62 | 373 | *Scaptotrigona postica* | 22/11/2019 | pollen | 06/12/2019 |
| 62 | 374 | *Scaptotrigona postica* | 22/11/2019 | pollen | 06/12/2019 |
| 62 | 375 | *Scaptotrigona postica* | 22/11/2019 | pollen | 06/12/2019 |
| 63 | 379 | *Scaptotrigona postica* | 22/11/2019 | pollen | 06/12/2019 |
| 63 | 380 | *Scaptotrigona postica* | 22/11/2019 | pollen | 06/12/2019 |
| 63 | 381 | *Scaptotrigona postica* | 22/11/2019 | pollen | 06/12/2019 |
| 64 | 383 | *Scaptotrigona postica* | 22/11/2019 | pollen | 06/12/2019 |
| 65 | 387 | *Scaptotrigona postica* | 22/11/2019 | pollen | 06/12/2019 |
| 65 | 388 | *Scaptotrigona postica* | 22/11/2019 | pollen | 06/12/2019 |
| 65 | 389 | *Scaptotrigona postica* | 22/11/2019 | pollen | 06/12/2019 |
| 66 | 392 | *Scaptotrigona postica* | 22/11/2019 | pollen | 06/12/2019 |
| 66 | 393 | *Scaptotrigona postica* | 22/11/2019 | pollen | 06/12/2019 |
| 67 | 1 | *Tetragonisca angustula* | 21/9/2019 | pollen | 6/12/2019 |
| 68 | 349 | *Tetragonisca angustula* | 22/11/2019 | pollen | 6/12/2019 |
| 68 | 350 | *Tetragonisca angustula* | 22/11/2019 | pollen | 6/12/2019 |
| 68 | 351 | *Tetragonisca angustula* | 22/11/2019 | pollen | 6/12/2019 |
| 69 | 3 | *Tetragonisca angustula* | 21/9/2019 | pollen | 06/12/2019 |
| 70 | 4 | *Tetragonisca angustula* | 21/9/2019 | pollen | 06/12/2019 |
| 70 | 356 | *Tetragonisca angustula* | 22/11/2019 | pollen | 06/12/2019 |
| 71 | 5 | *Tetragonisca angustula* | 21/9/2019 | pollen | 06/12/2019 |
| 72 | 6 | *Melipona rufiventris* | 21/9/2019 | pollen | 06/12/2019 |
| 73 | 7 | *Melipona rufiventris* | 21/9/2019 | pollen | 06/12/2019 |
| 74 | 8 | *Tetragonisca angustula* | 21/9/2019 | pollen | 06/12/2019 |
| 75 | 9 | *Scaptotrigona postica* | 21/9/2019 | pollen | 06/12/2019 |
| 76 | 10 | *Melipona rufiventris* | 21/9/2019 | pollen | 06/12/2019 |
| 77 | 11 | *Tetragonisca angustula* | 21/9/2019 | pollen | 06/12/2019 |
| 78 | 12 | *Tetragonisca angustula* | 21/9/2019 | pollen | 06/12/2019 |
| 79 | 13 | *Scaptotrigona postica* | 21/9/2019 | pollen | 06/12/2019 |
| 80 | 14 | *Scaptotrigona postica* | 21/9/2019 | pollen | 06/12/2019 |
| 81 | 15 | *Scaptotrigona postica* | 11/10/2019 | honey | 05/12/2019 |
| 81 | 16 | *Scaptotrigona postica* | 11/10/2019 | honey | 05/12/2019 |
| 81 | 17 | *Scaptotrigona postica* | 11/10/2019 | honey | 05/12/2019 |
| 82 | 21 | *Scaptotrigona postica* | 11/10/2019 | honey | 05/12/2019 |
| 82 | 22 | *Scaptotrigona postica* | 11/10/2019 | honey | 05/12/2019 |
| 82 | 23 | *Scaptotrigona postica* | 11/10/2019 | honey | 05/12/2019 |
| 83 | 24 | *Scaptotrigona postica* | 11/10/2019 | honey | 05/12/2019 |
| 83 | 25 | *Scaptotrigona postica* | 11/10/2019 | honey | 05/12/2019 |
| 83 | 26 | *Scaptotrigona postica* | 11/10/2019 | honey | 05/12/2019 |
| 84 | 33 | *Scaptotrigona postica* | 11/10/2019 | honey | 05/12/2019 |
| 84 | 34 | *Scaptotrigona postica* | 11/10/2019 | honey | 05/12/2019 |
| 84 | 35 | *Scaptotrigona postica* | 11/10/2019 | honey | 05/12/2019 |
| 85 | 43 | *Melipona rufiventris* | 11/10/2019 | honey | 05/12/2019 |
| 85 | 46 | *Melipona rufiventris* | 11/10/2019 | honey | 05/12/2019 |
| 86 | 47 | *Melipona rufiventris* | 11/10/2019 | honey | 05/12/2019 |
| 87 | 48 | *Tetragonisca angustula* | 11/10/2019 | honey | 05/12/2019 |
| 87 | 49 | *Tetragonisca angustula* | 11/10/2019 | honey | 05/12/2019 |
| 87 | 50 | *Tetragonisca angustula* | 11/10/2019 | honey | 05/12/2019 |
| 88 | 55 | *Tetragonisca angustula* | 11/10/2019 | honey | 05/12/2019 |
| 88 | 56 | *Tetragonisca angustula* | 11/10/2019 | honey | 05/12/2019 |
| 89 | 60 | *Tetragonisca angustula* | 11/10/2019 | honey | 05/12/2019 |
| 89 | 61 | *Tetragonisca angustula* | 11/10/2019 | honey | 05/12/2019 |
| 89 | 62 | *Tetragonisca angustula* | 11/10/2019 | honey | 05/12/2019 |
| 90 | 63 | *Tetragonisca angustula* | 11/10/2019 | honey | 05/12/2019 |
| 91 | 65 | *Melipona rufiventris* | 11/10/2019 | honey | 05/12/2019 |
| 91 | 66 | *Melipona rufiventris* | 11/10/2019 | honey | 05/12/2019 |
| 93 | 78 | *Scaptotrigona postica* | 11/10/2019 | honey | 13/12/2019 |
| 93 | 81 | *Scaptotrigona postica* | 11/10/2019 | honey | 13/12/2019 |
| 94 | 96 | *Scaptotrigona postica* | 29/10/2019 | honey | 13/12/2019 |
| 94 | 97 | *Scaptotrigona postica* | 29/10/2019 | honey | 13/12/2019 |
| 94 | 98 | *Scaptotrigona postica* | 29/10/2019 | honey | 13/12/2019 |
| 95 | 102 | *Scaptotrigona postica* | 29/10/2019 | honey | 13/12/2019 |
| 95 | 103 | *Scaptotrigona postica* | 29/10/2019 | honey | 13/12/2019 |
| 95 | 104 | *Scaptotrigona postica* | 29/10/2019 | honey | 13/12/2019 |
| 96 | 114 | *Scaptotrigona postica* | 29/10/2019 | honey | 13/12/2019 |
| 96 | 115 | *Scaptotrigona postica* | 29/10/2019 | honey | 13/12/2019 |
| 96 | 116 | *Scaptotrigona postica* | 29/10/2019 | honey | 13/12/2019 |
| 97 | 123 | *Melipona rufiventris* | 29/10/2019 | honey | 13/12/2019 |
| 97 | 124 | *Melipona rufiventris* | 29/10/2019 | honey | 13/12/2019 |
| 97 | 125 | *Melipona rufiventris* | 29/10/2019 | honey | 13/12/2019 |
| 98 | 132 | *Melipona rufiventris* | 29/10/2019 | honey | 13/12/2019 |
| 98 | 133 | *Melipona rufiventris* | 29/10/2019 | honey | 13/12/2019 |
| 98 | 134 | *Melipona rufiventris* | 29/10/2019 | honey | 13/12/2019 |
| 99 | 138 | *Tetragonisca angustula* | 29/10/2019 | honey | 13/12/2019 |
| 99 | 139 | *Tetragonisca angustula* | 29/10/2019 | honey | 13/12/2019 |
| 99 | 140 | *Tetragonisca angustula* | 29/10/2019 | honey | 13/12/2019 |
| 100 | 147 | *Tetragonisca angustula* | 29/10/2019 | honey | 13/12/2019 |
| 100 | 148 | *Tetragonisca angustula* | 29/10/2019 | honey | 13/12/2019 |
| 100 | 150 | *Tetragonisca angustula* | 29/10/2019 | honey | 13/12/2019 |
| 101 | 153 | *Melipona rufiventris* | 29/10/2019 | honey | 13/12/2019 |
| 101 | 154 | *Melipona rufiventris* | 29/10/2019 | honey | 13/12/2019 |
| 101 | 155 | *Melipona rufiventris* | 29/10/2019 | honey | 13/12/2019 |
| 102 | 162 | *Tetragonisca angustula* | 29/10/2019 | honey | 13/12/2019 |
| 102 | 163 | *Tetragonisca angustula* | 29/10/2019 | honey | 13/12/2019 |
| 102 | 164 | *Tetragonisca angustula* | 29/10/2019 | honey | 13/12/2019 |
| 103 | 165 | *Tetragonisca angustula* | 29/10/2019 | honey | 13/12/2019 |
| 103 | 166 | *Tetragonisca angustula* | 29/10/2019 | honey | 13/12/2019 |
| 103 | 167 | *Tetragonisca angustula* | 29/10/2019 | honey | 13/12/2019 |
| 104 | 171 | *Scaptotrigona postica* | 29/10/2019 | honey | 13/12/2019 |
| 104 | 172 | *Scaptotrigona postica* | 29/10/2019 | honey | 13/12/2019 |
| 104 | 173 | *Scaptotrigona postica* | 29/10/2019 | honey | 13/12/2019 |
| 105 | 195 | *Tetragonisca angustula* | 12/11/2019 | honey | 13/12/2019 |
| 105 | 196 | *Tetragonisca angustula* | 12/11/2019 | honey | 13/12/2019 |
| 105 | 197 | *Tetragonisca angustula* | 12/11/2019 | honey | 13/12/2019 |
| 106 | 201 | *Scaptotrigona postica* | 12/11/2019 | honey | 13/12/2019 |
| 106 | 210 | *Scaptotrigona postica* | 12/11/2019 | honey | 13/12/2019 |
| 106 | 211 | *Scaptotrigona postica* | 12/11/2019 | honey | 13/12/2019 |
| 107 | 207 | *Scaptotrigona postica* | 12/11/2019 | honey | 13/12/2019 |
| 107 | 208 | *Scaptotrigona postica* | 12/11/2019 | honey | 13/12/2019 |
| 107 | 209 | *Scaptotrigona postica* | 12/11/2019 | honey | 13/12/2019 |
| 108 | 213 | *Scaptotrigona postica* | 12/11/2019 | honey | 13/12/2019 |
| 108 | 214 | *Scaptotrigona postica* | 12/11/2019 | honey | 13/12/2019 |
| 108 | 215 | *Scaptotrigona postica* | 12/11/2019 | honey | 13/12/2019 |
| 109 | 216 | *Tetragonisca angustula* | 12/11/2019 | honey | 13/12/2019 |
| 109 | 217 | *Tetragonisca angustula* | 12/11/2019 | honey | 13/12/2019 |
| 109 | 218 | *Tetragonisca angustula* | 12/11/2019 | honey | 13/12/2019 |
| 110 | 86 | *Scaptotrigona postica* | 11/10/2019 | honey | 13/12/2019 |
| 110 | 229 | *Melipona rufiventris* | 12/11/2019 | honey | 13/12/2019 |
| 110 | 230 | *Melipona rufiventris* | 12/11/2019 | honey | 13/12/2019 |
| 110 | 231 | *Melipona rufiventris* | 12/11/2019 | honey | 13/12/2019 |
| 111 | 234 | *Tetragonisca angustula* | 12/11/2019 | honey | 13/12/2019 |
| 111 | 235 | *Tetragonisca angustula* | 12/11/2019 | honey | 13/12/2019 |
| 111 | 236 | *Tetragonisca angustula* | 12/11/2019 | honey | 13/12/2019 |
| 112 | 245 | *Tetragonisca angustula* | 12/11/2019 | honey | 13/12/2019 |
| 112 | 246 | *Tetragonisca angustula* | 12/11/2019 | honey | 13/12/2019 |
| 113 | 247 | *Tetragonisca angustula* | 12/11/2019 | honey | 13/12/2019 |
| 113 | 248 | *Tetragonisca angustula* | 12/11/2019 | honey | 13/12/2019 |
| 113 | 249 | *Tetragonisca angustula* | 12/11/2019 | honey | 13/12/2019 |
| 114 | 251 | *Melipona rufiventris* | 12/11/2019 | honey | 13/12/2019 |
| 114 | 252 | *Melipona rufiventris* | 12/11/2019 | honey | 13/12/2019 |
| 114 | 253 | *Melipona rufiventris* | 12/11/2019 | honey | 13/12/2019 |
| 115 | 257 | *Tetragonisca angustula* | 12/11/2019 | honey | 13/12/2019 |
| 115 | 258 | *Tetragonisca angustula* | 12/11/2019 | honey | 13/12/2019 |
| 115 | 259 | *Tetragonisca angustula* | 12/11/2019 | honey | 13/12/2019 |
| 116 | 264 | *Scaptotrigona postica* | 12/11/2019 | honey | 13/12/2019 |
| 116 | 265 | *Scaptotrigona postica* | 12/11/2019 | honey | 13/12/2019 |
| 116 | 266 | *Scaptotrigona postica* | 12/11/2019 | honey | 13/12/2019 |
| 117 | 268 | *Scaptotrigona postica* | 12/11/2019 | honey | 13/12/2019 |
| 117 | 277 | *Scaptotrigona postica* | 12/11/2019 | honey | 13/12/2019 |
| 117 | 278 | *Scaptotrigona postica* | 12/11/2019 | honey | 13/12/2019 |
| 117 | 280 | *Scaptotrigona postica* | 12/11/2019 | honey | 13/12/2019 |
| 118 | 281 | *Tetragonisca angustula* | 12/11/2019 | honey | 13/12/2019 |
| 118 | 282 | *Tetragonisca angustula* | 12/11/2019 | honey | 13/12/2019 |
| 118 | 283 | *Tetragonisca angustula* | 12/11/2019 | honey | 13/12/2019 |
| 119 | 274 | *Scaptotrigona postica* | 12/11/2019 | honey | 13/12/2019 |
| 119 | 275 | *Scaptotrigona postica* | 12/11/2019 | honey | 13/12/2019 |
| 119 | 276 | *Scaptotrigona postica* | 12/11/2019 | honey | 13/12/2019 |
| 120 | 292 | *Scaptotrigona postica* | 22/11/2019 | honey | 13/12/2019 |
| 120 | 293 | *Scaptotrigona postica* | 22/11/2019 | honey | 13/12/2019 |
| 120 | 294 | *Scaptotrigona postica* | 22/11/2019 | honey | 13/12/2019 |
| 121 | 298 | *Scaptotrigona postica* | 22/11/2019 | honey | 13/12/2019 |
| 121 | 300 | *Scaptotrigona postica* | 22/11/2019 | honey | 13/12/2019 |
| 122 | 307 | *Scaptotrigona postica* | 22/11/2019 | honey | 13/12/2019 |
| 122 | 308 | *Scaptotrigona postica* | 22/11/2019 | honey | 13/12/2019 |
| 122 | 309 | *Scaptotrigona postica* | 22/11/2019 | honey | 13/12/2019 |
| 123 | 319 | *Tetragonisca angustula* | 22/11/2019 | honey | 13/12/2019 |
| 123 | 320 | *Tetragonisca angustula* | 22/11/2019 | honey | 13/12/2019 |
| 123 | 321 | *Tetragonisca angustula* | 22/11/2019 | honey | 13/12/2019 |
| 124 | 362 | *Scaptotrigona postica* | 22/11/2019 | honey | 13/12/2019 |
| 124 | 363 | *Scaptotrigona postica* | 22/11/2019 | honey | 13/12/2019 |
| 124 | 372 | *Scaptotrigona postica* | 22/11/2019 | honey | 13/12/2019 |
| 125 | 342 | *Tetragonisca angustula* | 22/11/2019 | honey | 13/12/2019 |
| 125 | 344 | *Tetragonisca angustula* | 22/11/2019 | honey | 13/12/2019 |
| 125 | 345 | *Tetragonisca angustula* | 22/11/2019 | honey | 13/12/2019 |
| 126 | 346 | *Tetragonisca angustula* | 22/11/2019 | honey | 13/12/2019 |
| 126 | 347 | *Tetragonisca angustula* | 22/11/2019 | honey | 13/12/2019 |
| 126 | 348 | *Tetragonisca angustula* | 22/11/2019 | honey | 13/12/2019 |
| 127 | 359 | *Tetragonisca angustula* | 22/11/2019 | honey | 13/12/2019 |
| 127 | 360 | *Tetragonisca angustula* | 22/11/2019 | honey | 13/12/2019 |
| 127 | 367 | *Tetragonisca angustula* | 22/11/2019 | honey | 13/12/2019 |
| 128 | 364 | *Melipona rufiventris* | 22/11/2019 | honey | 13/12/2019 |
| 128 | 365 | *Melipona rufiventris* | 22/11/2019 | honey | 13/12/2019 |
| 128 | 366 | *Melipona rufiventris* | 22/11/2019 | honey | 13/12/2019 |
| 129 | 394 | *Scaptotrigona postica* | 10/12/2019 | pollen | 16/12/2019 |
| 130 | 395 | *Scaptotrigona postica* | 10/12/2019 | pollen | 16/12/2019 |
| 131 | 396 | *Tetragonisca angustula* | 10/12/2019 | pollen | 16/12/2019 |
| 132 | 397 | *Scaptotrigona postica* | 10/12/2019 | pollen | 16/12/2019 |
| 133 | 398 | *Tetragonisca angustula* | 10/12/2019 | pollen | 16/12/2019 |
| 134 | 399 | *Tetragonisca angustula* | 10/12/2019 | pollen | 16/12/2019 |
| 135 | 400 | *Tetragonisca angustula* | 10/12/2019 | pollen | 16/12/2019 |
| 136 | 401 | *Scaptotrigona postica* | 10/12/2019 | pollen | 16/12/2019 |
| 137 | 402 | *Tetragonisca angustula* | 10/12/2019 | pollen | 16/12/2019 |
| 138 | 403 | *Melipona rufiventris* | 10/12/2019 | pollen | 16/12/2019 |
| 139 | 404 | *Scaptotrigona postica* | 10/12/2019 | pollen | 16/12/2019 |
| 140 | 405 | *Melipona rufiventris* | 10/12/2019 | pollen | 16/12/2019 |
| 141 | 406 | *Tetragonisca angustula* | 10/12/2019 | pollen | 16/12/2019 |
| 142 | 407 | *Tetragonisca angustula* | 10/12/2019 | pollen | 16/12/2019 |
| 143 | 408 | *Scaptotrigona postica* | 10/12/2019 | pollen | 16/12/2019 |
| 144 | 409 | *Scaptotrigona postica* | 10/12/2019 | honey | 16/12/2019 |
| 145 | 410 | *Tetragonisca angustula* | 10/12/2019 | honey | 17/12/2019 |
| 146 | 411 | *Tetragonisca angustula* | 10/12/2019 | honey | 18/12/2019 |
| 147 | 412 | *Melipona rufiventris* | 10/12/2019 | honey | 19/12/2019 |
| 148 | 413 | *Scaptotrigona postica* | 10/12/2019 | honey | 20/12/2019 |
| 149 | 414 | *Scaptotrigona postica* | 10/12/2019 | honey | 21/12/2019 |
| 150 | 415 | *Scaptotrigona postica* | 10/12/2019 | honey | 22/12/2019 |
| 151 | 416 | *Tetragonisca angustula* | 10/12/2019 | honey | 23/12/2019 |
| 152 | 417 | *Tetragonisca angustula* | 10/12/2019 | honey | 24/12/2019 |
| 153 | 418 | *Melipona rufiventris* | 10/12/2019 | honey | 25/12/2019 |
| 154 | 419 | *Scaptotrigona postica* | 10/12/2019 | honey | 26/12/2019 |
| 155 | 420 | *Tetragonisca angustula* | 10/12/2019 | honey | 27/12/2019 |
| 156 | 421 | *Tetragonisca angustula* | 10/12/2019 | honey | 28/12/2019 |
| 157 | 422 | *Tetragonisca angustula* | 10/12/2019 | honey | 29/12/2019 |
| 158 | 423 | *Scaptotrigona postica* | 10/12/2019 | honey | 30/12/2019 |
| 159 | 284 | *Tetragonisca angustula* | 12/11/2019 | pollen | 16/12/2019 |
| 159 | 285 | *Tetragonisca angustula* | 12/11/2019 | pollen | 16/12/2019 |
| 159 | 286 | *Tetragonisca angustula* | 12/11/2019 | pollen | 16/12/2019 |
| 160 | 424 | *Melipona rufiventris* | 17/12/2019 | pollen | 18/12/2019 |
| 161 | 425 | *Melipona rufiventris* | 17/12/2019 | pollen | 18/12/2019 |
| 162 | 426 | *Tetragonisca angustula* | 17/12/2019 | pollen | 18/12/2019 |
| 163 | 427 | *Tetragonisca angustula* | 17/12/2019 | pollen | 18/12/2019 |
| 164 | 428 | *Tetragonisca angustula* | 17/12/2019 | pollen | 18/12/2019 |
| 165 | 429 | *Tetragonisca angustula* | 17/12/2019 | pollen | 18/12/2019 |
| 166 | 430 | *Tetragonisca angustula* | 17/12/2019 | pollen | 18/12/2019 |
| 167 | 431 | *Scaptotrigona postica* | 17/12/2019 | pollen | 18/12/2019 |
| 168 | 432 | *Scaptotrigona postica* | 17/12/2019 | pollen | 18/12/2019 |
| 169 | 433 | *Scaptotrigona postica* | 17/12/2019 | pollen | 18/12/2019 |
| 170 | 434 | *Scaptotrigona postica* | 17/12/2019 | pollen | 18/12/2019 |
| 171 | 435 | *Scaptotrigona postica* | 17/12/2019 | pollen | 18/12/2019 |
| 172 | 436 | *Scaptotrigona postica* | 17/12/2019 | pollen | 18/12/2019 |
| 173 | 437 | *Scaptotrigona postica* | 17/12/2019 | pollen | 18/12/2019 |
| 174 | 438 | *Melipona rufiventris* | 17/12/2019 | honey | 18/12/2019 |
| 175 | 439 | *Scaptotrigona postica* | 17/12/2019 | honey | 18/12/2019 |
| 176 | 440 | *Tetragonisca angustula* | 17/12/2019 | honey | 18/12/2019 |
| 177 | 441 | *Scaptotrigona postica* | 17/12/2019 | honey | 18/12/2019 |
| 178 | 442 | *Tetragonisca angustula* | 17/12/2019 | honey | 18/12/2019 |
| 179 | 443 | *Tetragonisca angustula* | 17/12/2019 | honey | 18/12/2019 |
| 180 | 444 | *Scaptotrigona postica* | 17/12/2019 | honey | 18/12/2019 |
| 181 | 445 | *Scaptotrigona postica* | 17/12/2019 | honey | 18/12/2019 |
| 182 | 446 | *Scaptotrigona postica* | 17/12/2019 | honey | 18/12/2019 |
| 185 | 449 | *Scaptotrigona postica* | 17/12/2019 | honey | 18/12/2019 |
| 186 | 450 | *Melipona rufiventris* | 17/12/2019 | honey | 18/12/2019 |
| 187 | 451 | *Scaptotrigona postica* | 17/12/2019 | honey | 18/12/2019 |

| **Table S2.** Amplicon sequences varieties (ASVs) with significant number of reads and their taxon matches. The IBGE column records presence/absence of taxa of any level in the IBGE flora (IBGE 2011). The RBS column records if/where species were recorded in the floristic survey (distances given from nests): G=garden (650m); N=nest plot (50m); I=inner pentagon plots (700m); O=outer pentagon plots (1500m); F1=near forest (630m); F2= distant forest (20XX); ? = automatically attributed to all reads not matched to species. The occurrence in honey or pollen is indicated by the bee species acronym in the relevant column: MR, *Melipona rufiventris*; SP, *Scaptotrigona postica* and TA, *Tetragonisca angustula*. Floral rewards to pollinators (pollen, nectar or oil) is presented as well as if the species is traditionally considered wind-pollinated. We assume all non-wind-pollinated are animal pollinated plants. | | | | | | | | | | | |
| --- | --- | --- | --- | --- | --- | --- | --- | --- | --- | --- | --- |
| **ASV** | **Higher Taxon** | **ASV Taxon matches** | **IBGE reserve flora** | **RBS floristic survey** | **origin** | **Occurrence honey** | **Occurrence pollen** | **Polen** | **Nectar** | **Oils** | **Wind pollinated** |
| 1 | Euphorbiaceae | *Acalypha sp* | yes | ? | native | MR | TA | yes | no |  | yes |
| 2 | Amaranthaceae | *Amaranthus viridis* | yes | no | weed or cultivated | SP | none | yes | yes |  | yes |
| 3 | Asteraceae | *Ambrosia polystachya* | yes | no | weed or cultivated | TA | none | yes | yes |  | no |
| 4 | Poaceae | *Anthaenantia lanata* | yes | no | native | none | TA | yes | no |  | yes |
| 5 | Moraceae | *Artocarpus heterophyllus* | yes | G | weed or cultivated | none | TA | yes | no |  | no |
| 6 | Asteraceae | *Asteraceae sp* | yes | ? | native | MR, SP, TA | TA | ? | ? |  | ? |
| 7 | Asteraceae | *Baccharis dracunculifolia* | yes | no | native | SP, TA | MR, SP, TA | yes | yes |  | no |
| 8 | Malpighiaceae | *Banisteriopsis sp* | yes | ? | native | none | TA | yes | no | yes | no |
| 9 | Fabaceae | *Bauhinia sp* | yes | ? | native | TA | none | yes | yes |  | no |
| 10 | Fabaceae | *Bauhinia ungulata* | no | no | native | TA | none | yes | yes |  | no |
| 11 | Chenopodiaceae | *Beta sp* | no | no | weed or cultivated | none | SP | yes | yes |  | no |
| 12 | Myrtaceae | *Blepharocalyx salicifolius* | yes | N,I,O,F1 | native | MR, SP, TA | MR, SP, TA | yes | no |  | no |
| 13 | Boraginaceae | *Borago officinalis* | no | no | weed or cultivated | SP | MR, SP, TA | yes | yes |  | no |
| 14 | Malpighiaceae | *Byrsonima basiloba* | yes | O | native | MR, TA | TA | yes | no | yes | no |
| 15 | Malpighiaceae | *Byrsonima crassifolia* | yes | no | native | TA | TA | yes | no | yes | no |
| 16 | Malpighiaceae | *Byrsonima laxiflora* | yes | O,F1 | native | TA | TA | no | no | yes | no |
| 17 | Malpighiaceae | *Byrsonima pachyphylla* | yes | N,I,O | native | SP, TA | MR, SP, TA | yes | no | yes | no |
| 18 | Malpighiaceae | *Byrsonima viminifolia* | yes | no | native | TA | TA | yes | no | yes | no |
| 19 | Myrtaceae | *Campomanesia pubescens* | yes | N,I,O | native | none | MR | yes | no |  | no |
| 20 | Cyperaceae | *Carex sp* | yes | ? | native | SP, TA | MR | yes | no |  | yes |
| 21 | Urticaceae | *Cecropia pachystachya* | yes | F1,F2 | native | none | TA | yes | yes? |  | yes |
| 22 | Rubiaceae | *Chiococca nitida* | yes | no | native | none | TA | yes | yes |  | no |
| 23 | Fabaceae | *Cicer sp* | no | no | weed or cultivated | none | SP | yes | yes |  | no |
| 24 | Clusiaceae | *Clusia criuva* | yes | no | native | SP, TA | SP, TA | yes | no |  | no |
| 25 | Combretaceae | *Combretaceae sp* | yes | ? | native | none | TA | yes | yes |  | no |
| 26 | Convolvulaceae | *Convolvulus sp* | no | ? | weed or cultivated | SP, TA | none | yes | yes |  | no |
| 27 | Fabaceae | *Copaifera langsdorffii* | yes | O,F1 | native | none | MR | yes | yes |  | no |
| 28 | Chrysobalanaceae | *Couepia grandiflora* | yes | no | native | SP | SP, TA | yes | yes |  | no |
| 29 | Asteraceae | *Crepis japonica* | yes | no | weed or cultivated | SP | none | yes | yes |  | no |
| 30 | Euphorbiaceae | *Croton abaitensis* | yes | I,O | native | TA | TA | yes | yes |  | no |
| 31 | Euphorbiaceae | *Croton conduplicatus* | no | no | native | MR, SP, TA | TA | yes | yes |  | no |
| 32 | Cucurbitaceae | *Cucurbita maxima* | yes | no | weed or cultivated | SP | none | yes | yes |  | no |
| 33 | Apiaceae | *Cuminum sp* | yes | ? | weed or cultivated | none | SP | no | Yes |  | no |
| 34 | Lythraceae | *Cuphea sp* | yes | ? | native | MR, SP, TA | TA | yes | yes |  | no |
| 35 | Cyperaceae | *Cyperus sp* | yes | ? | native | TA | none | yes | no |  | yes |
| 36 | Brassicaceae | *Diplotaxis sp* | no | no | weed or cultivated | none | MR | yes | yes |  | no |
| 37 | Dumortieraceae | *Dumortiera hirsuta* | no | no | native | none | MR, SP, TA | no | no |  | no |
| 38 | Boraginaceae | *Echium sp* | no | no | weed or cultivated | TA | none | no | yes |  | no |
| 39 | Myrtaceae | *Eucalyptus sp* | yes | no | weed or cultivated | MR, SP, TA | MR, SP, TA | yes | yes? |  | no |
| 40 | Myrtaceae | *Eugenia involucrata* | yes | N,I,O | native | TA | TA | yes | no |  | no |
| 41 | Myrtaceae | *Eugenia sp* | yes | ? | native | none | none | yes | no |  | no |
| 42 | Euphorbiaceae | *Euphorbia potentilloides* | yes | N,O | native | none | TA | yes | no |  | no |
| 43 | Fabaceae | *Fabaceae spp.* | yes | ? | native | TA | MR, SP | ? | ? |  | no |
| 44 | Fabaceae | *Glycine max* | no | no | weed or cultivated | TA | SP |  | yes |  | no |
| 45 | Nyctaginaceae | *Guapira graciliflora* | yes | N,I,O | native | MR, SP, TA | MR, SP, TA | yes | yes |  | no |
| 46 | Nyctaginaceae | *Guapira noxia* | yes | N,I,O | native | none | MR, SP |  | Yes |  | no |
| 47 | Araliaceae | *Hedera sp* | no | ? | weed or cultivated | SP | SP |  | yes |  | no |
| 48 | Chloranthaceae | *Hedyosmum brasiliense* | yes | F1,F2 | native | SP, TA | MR, SP, TA | yes | no |  | yes |
| 49 | Asteraceae | *Helianthus annuus* | yes | no | weed or cultivated | TA | none | yes | yes |  | no |
| 50 | Aquifoliaceae | *Ilex affinis* | yes | F2 | native | none | SP, TA | no | yes |  | no |
| 51 | Melastomataceae | *Leandra polystachya* | yes | O | native | MR, SP | MR, SP | yes | no |  | no |
| 52 | Liliopsida | *Liliopsida sp* | yes | ? | native | MR, SP | none |  | ? |  | ? |
| 53 | Euphorbiaceae | *Mabea fistulifera* | yes | O | native | MR, SP, TA | SP, TA | yes | yes |  | no |
| 54 | Malpighiaceae | *Malpighiaceae sp* | yes | ? | native | TA | TA |  | no | yes | no |
| 55 | Malpighiales | *Malpighiales sp* | yes | ? | native | none | TA |  | no | yes | no |
| 56 | Anacardiaceae | *Mangifera indica* | yes | F1 | weed or cultivated | none | TA | yes | yes |  | no |
| 57 | Euphorbiaceae | *Maprounea guianensis* | yes | N,I,O,F1,F2 | native | SP, TA | MR, SP, TA | yes | no |  | no |
| 58 | Sapindaceae | *Matayba guianensis* | yes | N,I,O,F1,F2 | native | MR, SP, TA | MR, SP, TA | yes | yes |  | no |
| 59 | Melastomataceae | *Melastomataceae sp* | yes | ? | native | none | SP, TA |  | no |  | no |
| 60 | Melastomataceae | *Miconia albicans* | yes | N,I,O,F1 | native | none | TA | yes | no |  | no |
| 61 | Melastomataceae | *Miconia hirtella* | yes | F1 | native | MR | MR, SP | yes | yes |  | no |
| 62 | Melastomataceae | *Miconia leucocarpa* | yes | O | native | MR, SP, TA | MR, SP | yes | no |  | no |
| 63 | Melastomataceae | *Miconia nervosa* | yes | F2 | native | MR | MR | yes | no |  | no |
| 64 | Melastomataceae | *Miconia stenostachya* | yes | ? | native | MR, SP, TA | MR, SP, TA | yes | no |  | no |
| 65 | Moraceae | *Moraceae sp* | yes | ? | native | MR, SP, TA | MR, SP, TA | ? | ? |  | no |
| 66 | Myrtaceae | *Myrcia guianensis* | yes | N,I,O | native | MR, SP | MR, SP, TA | yes | no |  | no |
| 67 | Myrtaceae | *Myrcia linearifolia* | yes | N,I,O | native | MR, SP, TA | MR, SP, TA | yes | no |  | no |
| 68 | Myrtaceae | *Myrcia myrtillifolia* | yes | N,I,O | native | SP, TA | SP, TA | yes | no |  | no |
| 69 | Myrtaceae | *Myrcia pinifolia* | yes | no | native | MR, SP, TA | MR, SP, TA | yes | no |  | no |
| 70 | Myrtaceae | *Myrcia pubescens* | yes | no | native | none | MR, SP | yes | no |  | no |
| 71 | Myrtaceae | *Myrcia splendens* | yes | I,O,F1,F2 | native | MR, SP, TA | MR, SP, TA | yes | no |  | no |
| 72 | Myrtaceae | *Myrcia tomentosa* | yes | N,I,F1 | native | SP | SP, TA | yes | no |  | no |
| 73 | Primulaceae | *Myrsine sp* | yes | ? | native | MR, SP, TA | SP, TA | yes | no |  | no |
| 74 | Primulaceae | *Myrsine umbellata* | yes | F1,F2 | native | SP, TA | SP, TA | yes | no |  | no |
| 75 | Myrtaceae | *Myrtaceae sp* | yes | ? | native | MR, SP, TA | MR, SP, TA | ? | no |  | no |
| 76 | Papaveraceae | *Papaver sp* | no | no | weed or cultivated | none | MR, SP | yes | no |  | no |
| 77 | Poaceae | *Paspalum notatum* | yes | G | weed or cultivated | SP | none |  | no |  | yes |
| 78 | Celastraceae | *Peritassa laevigata* | yes | ? | native | none | TA | yes | no |  | no |
| 79 | Asteraceae | *Picris sp* | no | no | weed or cultivated | SP, TA | none | no | yes |  | no |
| 80 | Pinaceae | *Pinus sp* | no | no | weed or cultivated | TA | SP | yes | no |  | yes |
| 81 | Piperaceae | *Piper aduncum* | yes | F1?F2? | native | MR, SP, TA | MR, SP, TA | yes | no |  | no |
| 82 | Piperaceae | *Piper divaricatum* | yes | no | native | none | TA | no | yes |  | no |
| 83 | Piperaceae | *Piper sp* | yes | ? | native | SP, TA | none | no | yes |  | no |
| 84 | Asteraceae | *Piptocarpha rotundifolia* | yes | N,I,O | native | none | MR | no | yes |  | no |
| 85 | Celastraceae | *Plenckia populnea* | yes | N,I,O | native | TA | SP |  | yes |  | no |
| 86 | Rosaceae | *Prunus sp* | yes | no | native | none | MR | yes | yes |  | no |
| 87 | Cyperaceae | *Rhynchospora exaltata* | yes | N | native | none | SP | yes | no |  | yes |
| 88 | Phyllanthaceae | *Richeria grandis* | yes | F1,F2 | native | MR, SP, TA | MR, SP, TA | yes | yes |  | no |
| 89 | Rosaceae | *Rosa chinensis* | yes | G | weed or cultivated | SP, TA | MR, SP | yes | no |  | no |
| 90 | Rosaceae | *Rosa gallica* | no | no | weed or cultivated | none | MR | yes | no |  | no |
| 91 | Rosaceae | *Rubus urticifolius* | yes | F1,F2 | native | MR, SP, TA | MR, SP, TA | yes | yes? |  | no |
| 92 | Sapindaceae | *Sapindaceae sp* | yes | ? | native | MR | MR, SP, TA | ? | ? |  | ? |
| 93 | Araliaceae | *Schefflera macrocarpa* | yes | N | native | none | SP, TA | yes | no |  | no |
| 94 | Euphorbiaceae | *Sebastiania brasiliensis* | yes | no | native | none | TA |  |  |  | no |
| 95 | Sapindaceae | *Serjania lethalis* | yes | I,O,F1,F2 | native | none | MR | no | yes |  | no |
| 96 | Brassicaceae | *Sinapis alba* | no | no | weed or cultivated | SP, TA | none |  |  |  | no |
| 97 | Loranthaceae | *Struthanthus/Psittacanthus sp* | yes | ? | native | MR, SP, TA | MR, SP, TA | yes | yes |  | no |
| 98 | Fabaceae | *Stryphnodendron sp* | yes | ? | native | MR, SP | MR, SP, TA | yes | yes? |  | no |
| 99 | Styracaceae | *Styrax ferrugineus* | yes | N,I,O | native | MR | MR | no | yes |  | no |
| 100 | Styracaceae | *Styrax sp* | yes | ? | native | MR | MR, SP | no | yes |  | no |
| 101 | Boraginaceae | *Symphytum sp* | no | no | weed or cultivated | TA | none |  |  |  | no |
| 102 | Myrtaceae | *Syzygium cumini* | yes | F1 | weed or cultivated | MR, SP, TA | MR, SP, TA | yes | yes |  | no |
| 103 | Myrtaceae | *Syzygium sp* | yes | ? | weed or cultivated | SP | SP, TA |  |  |  | no |
| 104 | Anacardiaceae | *Tapirira guianensis* | yes | I,O,F1 | native | MR, SP, TA | MR, SP, TA | yes | yes |  | no |
| 105 | Combretaceae | *Terminalia sp* | yes | ? | native | none | SP |  | yes |  | no |
| 106 | Anacardiaceae | *Toxicodendron succedaneum* | yes | no | weed or cultivated | SP, TA | SP, TA | yes | yes |  | no |
| 107 | Cannabaceae | *Trema micranthum* | yes | no | native | TA | TA |  |  |  | yes |
| 108 | Poaceae | *Urochloa brizantha* | yes | O | weed or cultivated | MR | none | yes | no |  | yes |
| 109 | Urticaceae | *Urtica sp* | no | no | weed or cultivated | TA | none |  | yes |  | yes |
| 110 | Fabaceae | *Vicia sp* | no | no | weed or cultivated | TA | none |  | yes |  | no |

| **Table S3.** 30 most frequent species in ASVs, their habitats in Savanna Biome and habit (tree, shrub, subshrub, climber, hemiparasite), presence in polen or honey and resource offered (P: pollen; N: nectar; O: oil; R: resin). Habitat data from floristic inventory in this study. Habit data from Flora & Funga do Brasil (2023). Floral reward data from literature cited | | | | | | |
| --- | --- | --- | --- | --- | --- | --- |
| Higher taxon | species | habitat | habit | Occurrence honey | Occurrence pollen | Floral reward |
| Asteraceae | *Baccharis dracunculifolia* | Savanna | shrub | SP, TA | ALL | PN |
| Myrtaceae | *Blepharocalyx salicifolius* | Savanna | tree, shrub | ALL | ALL | P |
| Malpighiaceae | *Byrsonima basiloba* | Savanna | shrub | MR, TA | TA | PO |
| Malpighiaceae | *Byrsonima pachyphylla* | Savanna | tree, shrub | SP, TA | ALL | PO |
| Clusiaceae | *Clusia criuva* | Forest | tree, shrub | SP, TA | SP, TA | PR |
| Euphorbiaceae | *Croton conduplicatus* | Savanna | shrub, subshrub | ALL | TA | PN |
| Myrtaceae | *Eucalyptus sp* | Cultivated | tree, shrub | ALL | ALL | PN |
| Nyctaginaceae | *Guapira graciliflora* | Savanna | tree, shrub | ALL | ALL | PN |
| Chloranthaceae | *Hedyosmum brasiliense* | Forest | tree, shrub | SP, TA | ALL | P |
| Melastomataceae | *Leandra polystachya* | Savanna | shrub, subshrub | MR, SP | MR, SP | P |
| Euphorbiaceae | *Mabea fistulifera* | Savanna | tree, shrub | ALL | SP, TA | PN |
| Euphorbiaceae | *Maprounea guianensis* | Savanna/Forest | tree | SP, TA | ALL | P |
| Sapindaceae | *Matayba guianensis* | Savanna, Forest | tree, shrub | ALL | ALL | PN |
| Melastomataceae | *Miconia hirtella* | Forest | tree, shrub | MR | MR, SP | PN |
| Melastomataceae | *Miconia leucocarpa* | Savanna | tree, shrub | ALL | MR, SP | P |
| Melastomataceae | *Miconia stenostachya* | Savanna | shrub | ALL | ALL | P |
| Myrtaceae | *Myrcia guianensis* | Savanna | tree, shrub, subshrub | MR, SP | ALL | P |
| Myrtaceae | *Myrcia linearifolia* | Savanna | shrub, subshrub | ALL | ALL | P |
| Myrtaceae | *Myrcia pinifolia* | Savanna | shrub | ALL | ALL | P |
| Myrtaceae | *Myrcia tomentosa* | Savanna, Forest | tree, shrub | SP | SP, TA | P |
| Primulaceae | *Myrsine sp* | ? | ? | ALL | SP, TA | P |
| Myrtaceae | *Myrtaceae sp* | ? | ? | ALL | ALL | ? |
| Piperaceae | *Piper aduncum* | Forest | tree, shrub | ALL | ALL | P |
| Phyllanthaceae | *Richeria grandis* | Forest | tree, shrub | ALL | ALL | PN |
| Rosaceae | *Rubus urticifolius* | Forest | climber, shrub, subshrub | ALL | ALL | PN |
| Loranthaceae | *Struthanthus/Psittacanthus sp* | ? | hemiparasite | ALL | ALL | PN |
| Fabaceae | *Stryphnodendron sp* | Savanna | ? | MR, SP | ALL | PN |
| Myrtaceae | *Syzygium cumini* | Forest (cultivated) | tree | ALL | ALL | PN |
| Anacardiaceae | *Tapirira guianensis* | Forest | tree | ALL | ALL | PN |
| Anacardiaceae | *Toxicodendron succedaneum* | Cultivated | tree | SP, TA | SP, TA | PN |
