# Supplementary figures and images for: Contrasting patterns of foraging behavior in neotropical stingless bees using pollen and honey metabarcoding
Source: Sci Rep. 2023 Sep 2;13:14474. doi: 10.1038/s41598-023-41304-0 (PMC10475120; doi:10.1038/s41598-023-41304-0)

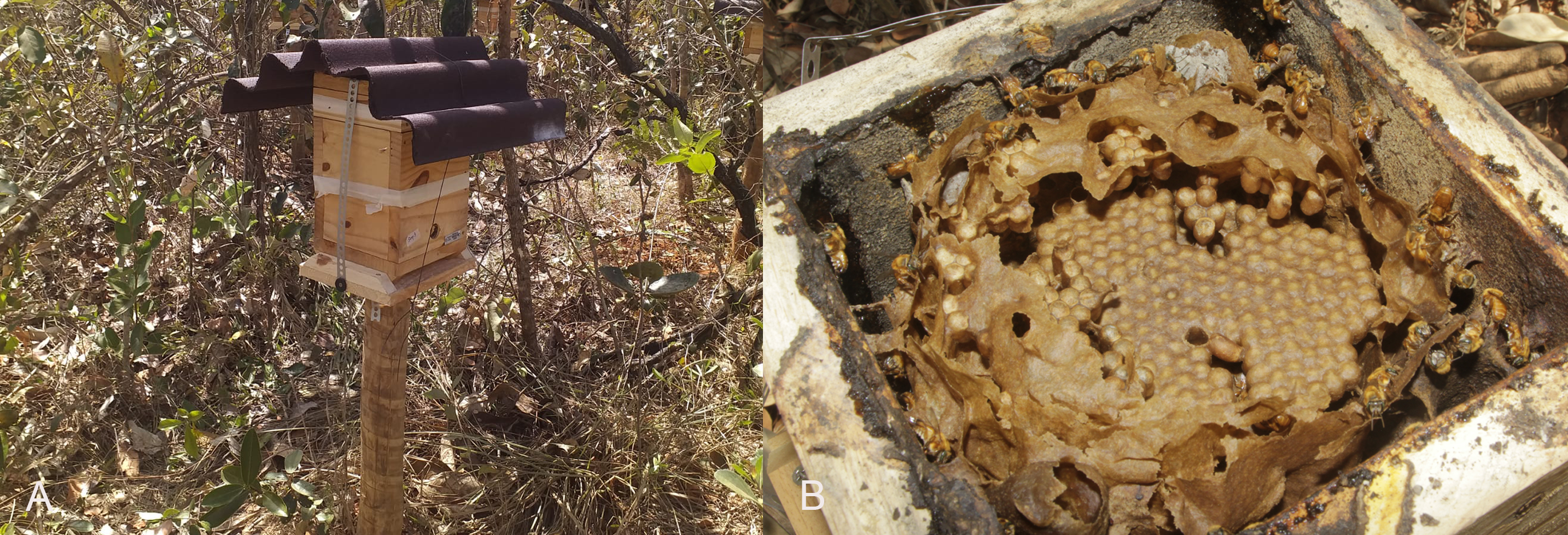

Supplement: Supplementary file 2 — Supplementary Figure S1. [file 41598_2023_41304_MOESM2_ESM.tif]

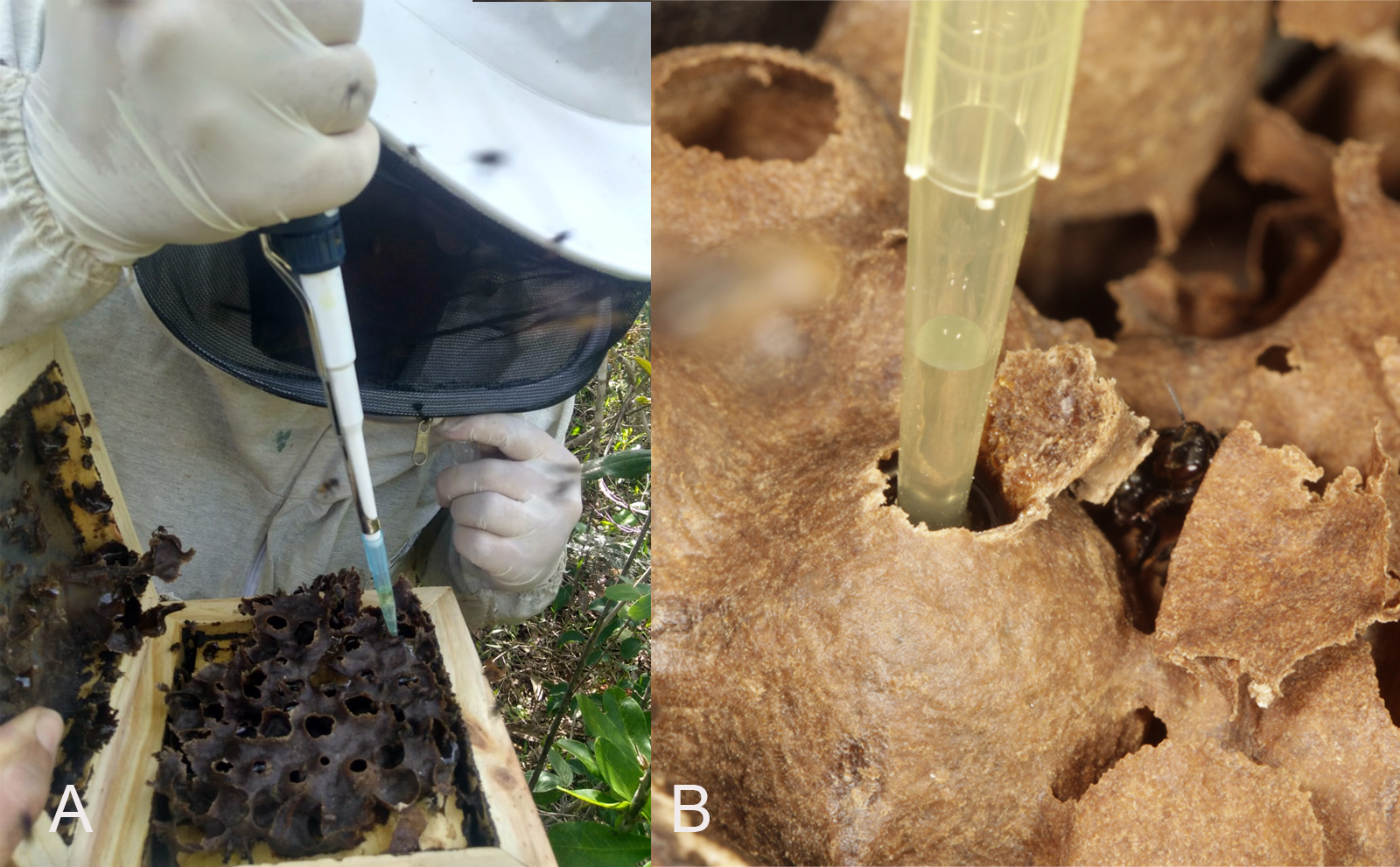

Supplement: Supplementary file 3 — Supplementary Figure S2. [file 41598_2023_41304_MOESM3_ESM.tif]
